# The Role of Illness Perception and Self-Efficacy in Determining Quality of Life among Cancer Patients

**DOI:** 10.3390/clinpract14020038

**Published:** 2024-03-19

**Authors:** Aisha Alhofaian, Marym M. Alaamri, Mariam Ahmed Abdalmajeed, Lama Saeed Wadaah, Leena Abdulaziz Aljuhani, Meral Ammar Amin, Afnan Tunsi, Ruba Alharazi

**Affiliations:** Faculty of Nursing, Medical Surgical Nursing Department, King Abdul Aziz University, P.O. Box 4929, Jeddah 22246, Saudi Arabia; mmalamri@kau.edu.sa (M.M.A.); myahyaabdalmajeed@stu.kau.edu.sa (M.A.A.); labdullahwadaah@stu.kau.edu.sa (L.S.W.); lahmedaljehani@stu.kau.edu.sa (L.A.A.); maamin@stu.kau.edu.sa (M.A.A.); atunsi@kau.edu.sa (A.T.); ralharazi@kau.edu.sa (R.A.)

**Keywords:** self-efficacy, illness perception, cancer, quality of life

## Abstract

Background: The quality of life for people with chronic illnesses like cancer has been shown to be significantly impacted by self-efficacy and perceptions of their illness. Objectives: This study investigates the relationship between cancer patients’ perceptions of their illness, their self-efficacy beliefs, and their quality of life. Method: Conducted from December 2022 to February 2023, this research involved 120 adults undergoing cancer treatment. We utilized the Illness Perception Questionnaire (IPQ), the Arabic version of the Cancer Behavioral Inventory Brief (CBI-B), and the Arabic EORTC QLQ-C30, alongside clinical data collection. Statistical analyses included Pearson correlation and descriptive statistics. Results: Breast cancer emerged as the most common type among participants. A positive correlation was found between self-efficacy and quality of life, as measured by the EORTC QLQ-C30, particularly in relation to symptom management. Interestingly, all dimensions of illness perception correlated with quality of life, except for control and concerns. Conclusions: The findings underscore the vital role of nurses and healthcare providers in aiding cancer patients to develop and utilize self-management strategies effectively. The study reveals that a patient’s capacity to manage their illness is significantly influenced by their confidence, understanding of their condition, and overall quality of life. Addressing these aspects can greatly enhance healthcare professionals’ contribution to improving the resilience and well-being of individuals battling cancer.

## 1. Introduction

The burden of cancer has increased over time in both industrialized and developing nations for a variety of complicated reasons. These include an aging and expanding population, swift socioeconomic development, and changes in the prevalence of risk factors associated with the disease [1]. Additionally, cultural influences significantly impact how patients perceive and manage their health. As Chou observed in 2019, varying cultural backgrounds can affect the selection of coping strategies and resources utilized in addressing illnesses [2].

A study conducted in San Francisco focused on the perspectives of Chinese cancer patients regarding their illness. The researcher used questionnaires to collect the data from a diverse Chinese patient population suffering from breast and colon cancer, totaling 159 participants. The findings concluded that patients with colon cancer had a significantly greater perception of the chronic nature and negative aspects of their cancer compared to those with breast cancer. On the other hand, patients with breast cancer demonstrated a significantly higher quality of life and confidence in making healthcare decisions [2].

In Poland, a study by [3] involving 202 women with noninvasive breast cancer treated surgically found a correlation between positive disease perception and enhanced quality of life, as well as reduced symptom intensity. This study highlights the concept of disease perception, where an individual’s response to illness is shaped by their unique experiences with the disease [3].

Conversely, a cross-sectional descriptive study in Korea, observed that while self-efficacy influenced quality of life, the patient’s view of their condition did not [4]. This study involved 46 participants and utilized the QLQ-C30 questionnaires to assess cancer patients’ quality of life. It also evaluated the effectiveness of the Maintain Function Scale in measuring self-efficacy among a new patient group, reaffirming the notion that self-efficacy can significantly impact clinical outcomes. The associations between illness awareness, self-care, self-efficacy, and self-care strategies and their impacts on quality of life were examined in a secondary data analysis on Chinese breast and colon cancer patients [2]. According to the study, Chinese cancer patients’ quality of life can be improved by increasing their ability to take care of themselves and exercise good self-control. When preparing for survival and educating patients, it is important to consider the differences in how patients perceive various cancer types.

Another researcher conducted a second study in which they looked at the connections between preexisting factors, mediating variables, and the quality of life of cancer patients [5]. A total of 314 cancer patients participated in this study. The results of this study concluded that anxiety, depression, and self-efficacy are significant factors in determining how cancer patients perceive their quality of life. There were mediating variables that either strengthened or increased the impact of the preceding factors on the severity of signs and the quality of life. The nurses should focus on advocating methods that boost self-efficacy [5].

Additionally, a Saudi Arabian study looked at the connection between self-efficacy and quality of life [6]. A total of 86 patients with gastrointestinal or breast cancer participated in this study. Their research revealed a strong relationship between emotional intelligence and self-efficacy. Additionally, only in the physical domain did the study find a statistically significant relationship between self-efficacy and quality of life. Additionally, there were found to be significant self-efficacy differences between men and women. Particularly, men with cancer are more self-effective than women with cancer. Compared to male cancer patients, female cancer patients have a better quality of life in terms of mental health status [6].

A qualitative phenomenological study was conducted in Ghana [7]. This study used semi-structured in-depth interviews to examine how women receiving breast cancer care perceived their illness and their coping mechanisms. According to the findings, there are three main factors that influence patients’ cognitive and emotional responses to their conditions: their understanding of breast cancer, their opinions regarding the causes of the illness, and the disease’s prognosis [7].

Finally, there has been a wide range of research on illness perceptions and self-efficacy, but most of it has been conducted in the United States or Africa, and there have been relatively few studies on the subject in Saudi Arabia. As a result, the main objective of this study was to assess the relationships among illness perceptions, self-efficacy, and quality of life among cancer patients.

## 2. Subjects and Methods

### 2.1. Study Designs

The researcher used a cross-sectional, descriptive, and correlational study to examine the relationship between illness perceptions, self-efficacy, and quality of life among cancer patients.

### 2.2. Setting

The researcher conducted this study at King Abdul Aziz University Hospital (KAUH) in Saudi Arabia, Jeddah, city. The KAUH is considered a big teaching hospital in Saudi Arabia with a 1067-bed capacity and more than 170 clinics, and it is operated by around 4000 healthcare experts and administrative personnel. It offers its services to both residents and citizens without distinction. The KAUH also provides a wide range of treatments for all types of complex cancers, including medical, surgical, and specialized cancer therapies.

### 2.3. Participants

In this study, a convenient sampling was used from KAUH departments. We used what is known as the convenient sampling strategy, which involves gathering data from the population that was accessible at the time of data collection. This approach ensures that the results of the study are accurately presented. From a data collection perspective, it is considered a quick and easy process. We recruited 120 cancer patients from King Abdul-Aziz University Hospital from January 2023 to April 2023. Age older than 18 years, the ability to read or speak Arabic, and a primary medical diagnosis of cancer were the inclusion criteria. It is important to note that patients in the study were receiving cancer treatment at that time. Patients who have cognitive impairment or any other severe chronic disease or severe psychiatric disturbance also met the exclusion criteria.

### 2.4. Instruments

A pre-made form was created to gather information about participant demographics, including age, gender, and type of cancer diagnosis. The Arabic version of the shortened Illness Perception Questionnaire was used to assess illness perception. It is the simplified form of the Illness Perception Questionnaire (IPQ). It consists of nine items, each of which is rated on a scale from 0 to 10 (where 10 corresponds to a poor perception of the disease). For each dimension, a greater perception of illness is indicated by a higher score. When Cronbach’s reliability = 0.77, it is both valid and reliable. It is made up of three parts: a cognitive illness representation, an emotional illness representation, and a representation of the understandability of the illness [8]. Numerous illnesses, including cancer, as well as minor illnesses, have been studied using the Brief Illness Perception Questionnaire.

Self-efficacy was measured with the Arabic version of the Cancer Behavioral Inventory-Brief. The 14 items on the CBI-B are scored on a Likert-type scale with a range of 1 to 9, or “not at all confident” (1) to “completely confident” (9). Higher scores suggest a greater level of coping self-efficacy. The CBI-B has subscales that measure involvement in medical care, coping with stress, managing effects, and preserving independence and a positive outlook [9]. There was sufficient evidence of reliability, as indicated by Cronbach’s alphas of ≥0.76.

The quality of life was measured with the Arabic version of the European Organization for Research and Treatment of Cancer Quality of Life Questionnaire Core 30 (EORTC QLQ-C30 [10]. The Arabic EORTC QLQ-C30 (Supplementary Materials) assesses the patient’s overall health status and quality of life, with each item converted from 0 to 100 points where possible. Greater scores on the symptom scales indicate worse symptoms, whereas greater scores on the functional subscales indicate better function. In its Arabic translation, this instrument was valid and trustworthy [10]. Its reliability has been assessed to be more than 0.70 for six of the nine subscales according to Cronbach’s alphas [10]. Cancer patients’ quality of life was measured using this scale.

### 2.5. Data Collection

After obtaining ethics approval, the participants were notified about participation in this study by a primary investigator. The consent form required the participants’ signatures. After the initial visit, the subjects were required to complete questionnaires about their sociodemographic information, perception of their illness, level of self-efficacy, and quality of life.

### 2.6. Statistical Analysis

For the current study’s statistical analyses, IBM SPSS Statistics 20 software was used. The continuous and categorical variables were described using descriptive statistics. It was analyzed using Pearson correlation, independent sample *t*-tests, and one-way ANOVA tests. The statistical level of significance in the current study was set at a *p*-value of less than 0.05. We acknowledge use of artificial intelligence (AI) ChatGPT versions GPT-3.5 for English editing of manuscript and no images were manipulated using AI. We estimated the sample size to be at least 120 participants according to a moderately small effect size of 0.25 and an alpha of 0.05 based on a previous study [11]. Therefore, the present sample size was sufficient to identify meaningful outcomes in this analysis.

### 2.7. Ethical Considerations

The researcher obtained ethical approval from the Research Ethical Committee of the Faculty of Nursing at King Abdul Aziz University (Reference No. 1F.12, approved date: 7 November 2022) and the Unit of Biomedical Ethics Research Committee at KAUH No (Reference No. 566-22, approved date: 13 December 2022) according to the guidelines (Declaration of Helsinki). Furthermore, no harm was imposed on the participants, and the researcher respected and protected the participants’ rights. Participation was voluntary and anonymous. Data would be confidential.

## 3. Results

Participants in the study had a mean age of 50.53 ± 3.63 years, and 67.5% of them were women, according to (Table 1). There were numerous primary cancer types, but breast cancer was the most prevalent (55.8%), followed by genitourinary cancer as well as GIT cancer (13.3%), which was the second most frequently reported type. Regarding the assessment of brief IPQ (Table 2), participants had a moderate perception of cancer with a total Brief-IPQ score of 43.4 ± 12.46. There was a low perception of cancer as a chronic disease that will last forever (mean 2.88 ± 2.8). However, high average scores on the “Treatment Control and Understanding scales” indicate that study participants strongly believe in the effectiveness of cancer treatment and have a good understanding of their disease. It is important to note that these patients generally perceive their personal control over their illness to be much less than their ability to control it through medical means.

Moreover, the average scores on the CBI-B and EORTC QLQ-C30 scales were 87.56 ± 24.56 and 61.48 ± 14.55, respectively (Table 2). The results of Table 3 and Table 4 demonstrate that there was no statistically significant correlation between the mean IPQ or CBI-B scores and the participant demographics or cancer type.

In addition, several variables are listed in (Table 5) together with the EORTC QLQ-C30 scores, the statistical test that was performed, and the *p*-values. For example, the mean score among women is 61.06 with a standard deviation of 14.62, and for men, it is 62.35 with a standard deviation of 14.57. The *p*-value for the male–female comparison was 0.649, indicating no statistically significant difference between males and females, as the *p*-value was greater than the normal alpha level of 0.05.

For cancer types, there were different mean scores listed. For example, lung cancer patients scored 76.42 with a standard deviation of 17.79. A one-way ANOVA test was used to analyze the cancer type variable with a *p*-value of 0.032, indicating a statistically significant difference in the EORTC QLQ-C30 scores for cancer types between at least two groups because the *p*-value is less than 0.05.

A significant positive correlation between the CBI-B scale and the EORTC QLQ-C30 (functional subscale) was discovered (r = 0.25, *p*-value = 0.006), as shown in Table 6. As a result, better quality of life was linked to stronger beliefs that one could play a part in managing the illness, taking part in medical treatment, managing stress, and managing effects. However, there was a significant negative correlation between the CBI-B scale and the EORTC QLQ-C30 (symptom subscale) and the total scale (r = −0.29, *p*-value = 0.001).

The consequences, timeline, identity, and emotional response subscales of the IPQ scale and the EORTC QLQ-C30 also showed a significant positive correlation (*p* ≤ 0.05), while the EORTC QLQ-C30 and the IPQ scale’s treatment control and understanding subscales had a statistically significant negative correlation (*p* ≤ 0.05).

## 4. Discussion

The impact of self-efficacy and illness perceptions on the quality of life in individuals with chronic illnesses, such as cancer, has been increasingly recognized. Self-efficacy, which refers to an individual’s belief in their ability to exert control over their own functioning and events that affect their lives, plays a crucial role. Patients who possess a strong sense of self-efficacy tend to have a greater sense of control and actively engage in managing their symptoms [12]. This proactive behavior can lead to improved physical health and subjective well-being. The challenges posed by living with cancer can significantly affect a patient’s quality of life, as noted by [13]. Therefore, the primary objective of this study was to explore the interrelationships between illness perceptions, self-efficacy, and quality of life among cancer patients. Initially, there was a strong correlation between all aspects of illness perception and quality of life, except for personal control and concerns. However, only the identity, consequences, timeline, and emotional response subscales of the IPQ scale demonstrated positive correlations (*p* = 0.05) in the current study. The identity aspect, which relates to the symptoms patients associate with their cancer, seems to have a significant impact. Patients who recognize and understand their symptoms may be better equipped to seek appropriate care and manage their condition effectively. The consequences and timeline dimensions suggest that patients who acknowledge the severity and chronic nature of cancer may be more motivated to engage in long-term health management, which could positively influence their quality of life. To put it another way, the perception of cancer as a chronic condition (timeline) might lead patients to adopt long-term coping strategies, positively affecting their quality of life. Additionally, understanding how the perception of severe consequences impacts patients’ mental health and daily living can be crucial. These findings align with previous studies on illness perception among cancer patients, indicating that those with negative perceptions experience a detrimental impact on their quality of life [14,15]. Therefore, the patients who hold negative perceptions about their cancer, specifically regarding their feelings and the perceived impact, should be recognized and supported by the oncology nurses during the early stages. This can be achieved by asking the patients about their emotional well-being and beliefs about their disease.

The patients’ scores varied widely. The mean scores for illness perception and self-efficacy were 43.4 ± 12.46 and 87.56 ± 24.56. According to the strategy of the health action process, self-efficacy assessment measures the degree to which a person believes that they can manage the current circumstance, which is consistent with the strong link between self-efficacy and quality of life in terms of functional subscale.

The EORTC QLQ-C30’s functional subscale and the EORTC QLQ-C30’s symptom subscale showed a positive and negative correlation between self-efficacy for coping with cancer, respectively. Higher self-efficacy likely leads to more proactive management of the illness and better handling of daily activities, thereby improving functional status [5]. This relationship suggests that interventions aimed at increasing self-efficacy could directly impact patients’ ability to maintain their daily routines and manage symptoms effectively. It would be beneficial to explore in detail how different levels of self-efficacy correlate with specific aspects of functional status. These findings are consistent with research on the quality of life of cancer patients, which shows a link between higher levels of self-efficacy and better health and quality of life [4,6]. The importance of self-efficacy in sustaining daily activities for quality of life adds new and important clinical data. These findings also support the original validation of the scale as well as more recent studies that show the beneficial effects of self-efficacy on the quality of life of cancer patients, people with chronic disabilities, and those who provide care for them.

In conclusion, this study offers valuable insights into the interrelationships between illness perceptions, self-efficacy, and quality of life among cancer patients. Our findings reveal significant correlations between various aspects of illness perception and quality of life, highlighting the crucial role of patients’ mental frameworks in managing their condition. The study emphasizes the importance of understanding and addressing the psychological aspects of cancer care, particularly in enhancing patients’ self-efficacy and coping strategies. To our knowledge, this finding is reported for the first time in Saudi Arabia, which is considered a strength of the present study.

Although the research team has strived to maintain methodological rigor in the current study, it has some limitations. For instance, the use of convenience sampling may affect the generalizability of the study findings. Moreover, the focus on a single medical center may not accurately represent the views of all cancer patients in Saudi Arabia. Nonetheless, our findings can act as a starting point for future research that examines how cancer patients’ self-efficacy and illness perceptions affect their overall health in more diverse settings. Finally, the inclusion of a broader range of cancer types and the application of more comprehensive statistical analyses, such as regression, would further elucidate the dynamics between illness perception, self-efficacy, and quality of life.

The current study sheds light on how cancer patients’ quality of life is impacted by illness perception and self-efficacy. Because illness perceptions can be altered, cancer patients may be able to adopt more adaptive coping mechanisms by altering their illness perceptions. Therefore, our findings may assist in the development of specific cancer patients’ support programs that provide coping mechanisms and structurally consider how they perceive their condition. More specifically, interventions could target reducing emotional issues and distress by addressing the illness’s perceived duration and the impact it has on patients’ quality of life (and removing any misconceptions). According to our findings, cancer patients may benefit more from support to reduce their condition’s perceived threat than from assistance to reinforce their control beliefs. Patients with cancer may receive the resources they require to manage their condition, which may enhance their quality of life.

## 5. Conclusions

The results of this study provide valuable insights into the complex interplay between illness perceptions, self-efficacy, and quality of life among cancer patients. The findings also highlight the substantial influence of patients’ perceptions of their illness and their beliefs in their ability to cope, which will all affect overall quality of life. Particularly, some aspects of illness perception, such as identity, consequences, timeline, and emotional response, demonstrated positive correlations with quality of life, suggesting that how patients view their illness can significantly impact their well-being.

Although certain aspects of illness perception correlated with quality of life, other aspects like personal control and concerns did not show a strong link, implying the complex nature of these relationships. This emphasizes the importance of tailored intervention approaches in cancer care, where psychological and emotional needs are addressed alongside physical health.

## Figures and Tables

**Table 1:** Distribution of studied participants according to their demographics and type of cancer (No.: 120).

Variable	No. (%)
Age	50.53 ± 13.63
Gender	
Female	81 (67.5)
Male	39 (32.5)
Cancer type	
Blood cancer	5 (4.2)
Breast cancer	67 (55.8)
Genitourinary cancer	16 (13.3)
GIT cancers	16 (13.3)
Lung cancer	7 (5.8)
Lymphoma	3 (2.5)
Mixed cancers	2 (1.7)
Skin cancer	3 (2.5)
Thyroid cancer	1 (0.8)

**Table 2:** Mean and SD of the used Illness Perception Questionnaire (IPQ) and its subscales, Cancer Behavioral Inventory-Brief, and the European Organization for Research and Treatment of Cancer Quality of Life Questionnaire Core 30 (EORTC QLQ-C30) (No.: 120).

Scale	Mean SD
Illness Perception Questionnaire (IPQ)	43.4 ± 12.46
Illness Perception Questionnaire (IPQ) subscales	
Consequences	4.83 ± 2.76
Timeline	2.88 ± 2.8
Personal	6.66 ± 2.68
Treatment control	7.07 ± 3.16
Identity	4.22 ± 2.97
Concern	5.26 ± 3.16
Understanding	7.38 ± 3.3
Emotional response	5.13 ± 3.29
Cancer Behavioral Inventory-Brief. The CBI-B	87.56 ± 24.56
European Organization for Research and Treatment of Cancer Quality of Life Questionnaire Core 30 (EORTC QLQ-C30)	61.48 ± 14.55

**Table 3:** Relationship between the mean IPQ scores and participants’ demographics and type of cancer (No.: 120).

Variable	IPQ	Test	*p*-Value
Gender		1.83 *	0.069
Female	41.97 ± 13.39		
Male	46.38 ± 9.74		
Cancer type		1.26 **	0.27
Blood cancer	50.8 ± 9.36		
Breast cancer	42.67 ± 13.37		
Genitourinary cancer	45.81 ± 13.01		
GIT cancers	45.31 ± 9.54		
Lung cancer	47.71 ± 7.84		
Lymphoma	32.33 ± 11.93		
Mixed cancers	29.5 ± 7.77		
Skin cancer	38.66 ± 9.07		
Thyroid cancer	32 ± 7.63		

N.B.: * = independent sample *t*-test. ** = one-way ANOVA test.

**Table 4:** Relationship between the mean CBI-B scores and participants’ demographics and type of cancer (No.: 120).

Variable	CBI-B	Test	*p*-Value
Gender		0.9	0.368
Female	88.97 ± 23.8		
Male	84.64 ± 26.13		
Cancer type		1.52	0.158
Blood cancer	91.6 ± 21.69		
Breast cancer	90.49 ± 22		
Genitourinary cancer	82.43 ± 32.47		
GIT cancers	93.62 ± 20.86		
Lung cancer	65.85 ± 27.64		
Lymphoma	75.66 ± 6.5		
Mixed cancers	98.5 ± 24.74		
Skin cancer	77 ± 40.36		
Thyroid cancer	54 ± 7.81		

**Table 5:** Relationship between the mean EORTC QLQ-C30 scores and participants’ demographics and type of cancer (No.: 120).

Variable	EORTC QLQ-C30	Test	*p*-Value
Gender		0.45	0.649
Female	61.06 ± 14.62		
Male	62.35 ± 14.57		
Cancer type		2.2	0.032
Blood cancer	67.2 ± 22.52		
Breast cancer	61.22 ± 13.72		
Genitourinary cancer	56.68 ± 14.62		
GIT cancers	59.87 ± 10.17		
Lung cancer	76.42 ± 17.79		
Lymphoma	61 ± 15.52		
Mixed cancers	46 ± 5.65		
Skin cancer	58.33 ± 7.57		
Thyroid cancer	90 ± 13.64		

**Table 6:** Pearson correlation analysis between the IPQ, CBI-B, and EORTC QLQ-C30 (functional and symptom and total scale): correlation with patients’ age and between each other.

Variable	IPQ	*p*-Value
	r	
Age	0.03	0.716
CBI-B	0.13	0.139
EORTC QLQ-C30 (functional subscale)	0.17	0.05
EORTC QLQ-C30 (symptom subscale)	0.12	0.165
EORTC QLQ-C30	0.16	
	CBI-B	
Age	0.12	0.177
EORTC QLQ-C30 (functional subscale)	0.25	0.006
EORTC QLQ-C30 (symptom subscale)	−0.29	0.001
EORTC QLQ-C30	−0.29	0.001
	EORTC QLQ-C30 (functional subscale)	
Age	0.03	0.69
	EORTC QLQ-C30 (symptom subscale)	
Age	0.13	0.147
	EORTC QLQ-C30	
Age	0.08	0.333
Illness Perception Questionnaire (IPQ) subscales		
Consequences	0.32	<0.001
Timeline	0.18	0.044
Personal	−0.13	0.152
Treatment control	−0.25	0.006
Identity	0.23	0.009
Concern	0.16	0.081
Understanding	−0.19	0.03
Emotional response	0.29	0.001

## Data Availability

The data presented in this study are available on request from the corresponding author due to privacy.

## Supplementary Materials

The following supporting information can be downloaded at: https://www.mdpi.com/article/10.3390/clinpract14020038/s1, Scale S1: EORTC QLQ-C30, Scale S2: Brief Illness Perception Questionnaire-Arabic Language, Scale S3: The Cancer Behavioral Inventory-Brief Arabic and English Version in Lebanon.
