# Supplementary material for: The Role of Illness Perception and Self-Efficacy in Determining Quality of Life among Cancer Patients
Source: Clin Pract. 2024 Mar 19;14(2):498–507. doi: 10.3390/clinpract14020038 (PMC10961753; doi:10.3390/clinpract14020038)
Supplement: Supplementary file 1 [file clinpract-14-00038-s001.zip › clinpract-2884031-supplementary.pdf]

EORTC QLQ-C30 (Version 3)

نحن مهتمون بمعرفة بعض المعلومات عنك و عن صحتك. الرجاء الإجابة بنفسك عن كل من الأسئلة التالية و ذلك بوضع دائرة حول الإجابة الأكثر ملائمة لك. لا يوجد جواب "صحيح" أو "خطأ". جميع المعلومات ستعامل بسرية تامة.

الرجاء إدخال الحروف الأولى من اسمك: |_|_|_|_|

تاريخ ميلادك (اليوم، الشهر، السنة): |_|_|_|_|_|_|_|_|

تاريخ اليوم (اليوم، الشهر، السنة): 31|_|_|_|_|_|_|_|_|

		إطلاقًا	قليلاً	بما فيه الكفاية	كثيرًا جدًا
.1	هل لديك صعوبة في بذل مجهود جسدي شاق (متعب) مثل حمل كيس مشتريات ثقيل أو حقيبة؟	1	2	3	4
.2	هل لديك صعوبة بالمشي لمسافة طويلة؟	1	2	3	4
.3	هل لديك صعوبة بالمشي لمسافة قصيرة خارج البيت؟	1	2	3	4
.4	هل تحتاج للبقاء في السرير أو الكرسي خلال اليوم؟	1	2	3	4
.5	هل تحتاج للمساعدة في الأكل أو ارتداء الملابس أو الاغتسال أو استخدام المرحاض؟	1	2	3	4

	**خلال الأسبوع الماضي:**	إطلاقًا	قليلاً	بما فيه الكفاية	كثيرًا جدًا
.6	هل كنت محدودًا/ مقيدًا عند القيام بعملك أو نشاطات يومية أخرى؟	1	2	3	4
.7	هل كنت محدودًا/ مقيدًا في ممارسة هواياتك أو نشاطات في اوقات الفراغ؟	1	2	3	4
.8	هل شعرت بضيق بالنفس؟	1	2	3	4
.9	هل شعرت بأي ألم؟	1	2	3	4
.10	هل كنت بحاجة للراحة؟	1	2	3	4
.11	هل عانيت من مشاكل في النوم (أرق/ صعوبة في النوم/ نوم متقطع)؟	1	2	3	4
.12	هل شعرت بالضعف؟	1	2	3	4
.13	هل فقدت شهيتك للطعام (القدرة على الاكل)؟	1	2	3	4
.14	هل شعرت بالغثيان (اللعيان)؟	1	2	3	4
.15	هل تقيأت؟	1	2	3	4
.16	هل عانيت من إمساك؟	1	2	3	4

انتقل إلى الصفحة التالية من فضلك

	خلال الأسبوع الماضي:	إطلاقًا	قليلاً	بما فيه الكفاية	كثيرًا جدًا
17.	هل كان لديك إسهال؟	1	2	3	4
18.	هل كنت متعبًا؟	1	2	3	4
19.	هل عانيت من ألم أثّر سلبيًا على نشاطاتك اليومية؟	1	2	3	4
20.	هل كان لديك صعوبة بالتركيز في بعض الأمور مثل قراءة الجريدة أو مشاهدة التلفاز؟	1	2	3	4
21.	هل شعرت بالتوتر؟	1	2	3	4
22.	هل شعرت بالقلق؟	1	2	3	4
23.	هل شعرت بالإنزعاج؟	1	2	3	4
24.	هل شعرت بإكتئاب ؟	1	2	3	4
25.	هل كانت لديك صعوبة بتذكر الأشياء؟	1	2	3	4
26.	هل حالتك الجسدية أو علاجك الطبي أثّر سلبيًا على حياتك العائلية؟	1	2	3	4
27.	هل حالتك الجسدية أو علاجك الطبي أثّر سلبيًا على حياتك الاجتماعية؟	1	2	3	4
28.	هل حالتك الجسدية أو علاجك الطبي أديا إلى مشاكل مالية؟	1	2	3	4

في الأسئلة التالية الرجاء الإشارة بدائرة حول الأرقام بين 1 - 7 الاكثر ملائمة لك

29. كيف تُقيّم صحتك عمومًا خلال الأسبوع الماضي؟

1	2	3	4	5	6	7
سيء جدًا						ممتاز

30. كيف تُقيّم /جودة حياتك عمومًا/ مستوى حياتك عمومًا خلال الأسبوع الماضي؟

1	2	3	4	5	6	7
سيء جدًا						ممتاز

© حقوق الطبع 1995، EORTC Quality of Life Group، جميع الحقوق محفوظة. النسخة 3.0

Brief Illness Perception Questionnaire-Arabic Language Version in Lebanon

Lydia Rabbaa Khabbaz

إلى أي حد يؤثّر مرضكم على حياتكم؟

صفر	1	2	3	4	5	6	7	8	9	10
لا يؤثّر عليها أبداً										يؤثّر عليها بشدّة

كم تظنون أنّ مرضكم سيدوم؟

صفر	1	2	3	4	5	6	7	8	9	10
لمدة قليلة جداً										إلى الأبد

كيف تقيّمون سيطرتكم على المرض؟

صفر	1	2	3	4	5	6	7	8	9	10
لا سيطرة على الإطلاق										سيطرة هائلة

كم تظنون أن علاجكم يمكن أن يساعدكم؟

صفر	1	2	3	4	5	6	7	8	9	10
لا يساعدني على الإطلاق										مفيد الى اقصى درجة

الى اي درجة تشعرون بعوارض مرضكم؟

صفر	1	2	3	4	5	6	7	8	9	10
لا عوارض على الإطلاق										عوارض حادة جدا

إلى أي حد يسبب لكم مرضكم القلق؟

صفر	1	2	3	4	5	6	7	8	9	10
لا قلق على الإطلاق										قلق هائل

كيف تقيّمون فهمكم لمرضكم؟

صفر	1	2	3	4	5	6	7	8	9	10
لا أفهمه على الإطلاق										أفهمه بوضوح فائق

كم يؤثّر عليكم مرضكم على الصعيد العاطفي؟ (مثلاً يثير غضبكم، يخيفكم، يحزنكم أو يسبب لكم الاحباط ...)؟

صفر	1	2	3	4	5	6	7	8	9	10
لا أتأثر على الإطلاق										أتأثر بدرجة هائلة

يرجى منكم أن تذكروا، بترتيب الأهمية، الأسباب الثلاثة التي أدت (برأيكم) إلى مرضكم. الأسباب الأكثر أهمية بالنسبة إلي:

1.
2.
3.

The Cancer Behavioral Inventory-Brief Arabic in English

Please complete the following:

1- Demographics:
Age: ___________ Gender: ___________ Type of Cancer: ____________

2- Questionnaire about cancer patient behavior:
This questionnaire consists of things a person might do when receiving cancer treatment. We are interested in your level of confidence for performance of these things. Be sure that these ratings precisely reflect your confidence whether you did it before or not. Therefore, the rating will reflect your level of confidence in the performance of these things in the present (or in the near future). Please read each numbered item, then rate your level of confidence in the performance of this behavior. Put a circle around the number in the rating scale. If you circle number "1", you mean that you are not at all confident in performing this behavior. If you circle number "9", you mean that you are totally confidant in performing this behavior. The numbers in the middle of the scale show that you are somewhat confidant in performing this behavior. Please rate all items. If you are not sure about certain items, please provide the best rating you can.

	Not Confidant at all			Somewhat Confidant			Totally Confidant		
1- Maintain self-reliance	1	2	3	4	5	6	7	8	9
	Not Confidant at all			Somewhat Confidant			Totally Confidant		
2- Maintain positive attitude	1	2	3	4	5	6	7	8	9
	Not Confidant at all			Somewhat Confidant			Totally Confidant		
3- Maintain sense of humor	1	2	3	4	5	6	7	8	9
	Not Confidant at all			Somewhat Confidant			Totally Confidant		
4- Expressing feelings about the disease	1	2	3	4	5	6	7	8	9
	Not Confidant at all			Somewhat Confidant			Totally Confidant		
5- Talk about things on my mind at times	1	2	3	4	5	6	7	8	9
	Not Confidant at all			Somewhat Confidant			Totally Confidant		
6- Maintain activities (work, home, hobbies, social)	1	2	3	4	5	6	7	8	9
	Not Confidant at all			Somewhat Confidant			Totally Confidant		
7- Try to be calm during treatment and avoid frightening ideas	1	2	3	4	5	6	7	8	9
	Not Confidant at all			Somewhat Confidant			Totally Confidant		
8- Participate actively in treatment decisions	1	2	3	4	5	6	7	8	9
	Not Confidant at all			Somewhat Confidant			Totally Confidant		
9- Ask physician questions	1	2	3	4	5	6	7	8	9
	Not Confidant at all			Somewhat Confidant			Totally Confidant		
10- Seek support from others such as family, friends, groups, and organizations	1	2	3	4	5	6	7	8	9
	Not Confidant at all			Somewhat Confidant			Totally Confidant		
11- Share my fears and concerns with others	1	2	3	4	5	6	7	8	9
	Not Confidant at all			Somewhat Confidant			Totally Confidant		
12. Control nausea and vomiting (whether you have had it before or not)	1	2	3	4	5	6	7	8	9
	Not Confidant at all			Somewhat Confidant			Totally Confidant		
13. Cope with physical difficulties	1	2	3	4	5	6	7	8	9
	Not Confidant at all			Somewhat Confidant			Totally Confidant		
14. Try to maintain calm during waiting for appointment for one hour or more	1	2	3	4	5	6	7	8	9

الرجاء كتابة مايلي:

1- معلومات عامه

العمر-------- **الجنس----------** **نوع السرطان----------------------------**

2- استبيان سلوك مريض السرطان

يحتوي هذا الاستبيان على امور قد يقوم بها الشخص عندما يتلقى علاج السرطان
نحن مهتمون بتقييمك لمدى ثقتك في امكانية انجازك لهذه الامور.تأكد من ان التقييمات تعكس بدقه مدى ثقتك سواء قمت ام لم تقم بها في السابق
وبالتالي فان هذه التقييمات تعكس مدى ثقتك في امكانية انجازك لهذه الامور حاليا (او في المستقبل القريب
الرجاء قراءة كل عنصر مرقم, ثم قيم هذا العنصر حول مدى ثقتك في امكانية انجاز هذا السلوك. ضع دائره على الرقم الموجود في مقياس التقييم اذا وضعت دائره على رقم1 فانك توضح انك غير واثق تماما من انه يمكنك انجاز هذا التصرف. اذا وضعت دائره على رقم 9 فانك توضح انك واثق تماما من انه يمكنك انجاز هذا التصرف . اما الارقام التي فو وسط مقياس التقييم فتشير الى انك واثق نوعا ما في امكانية قيامك بهذا السوك الرجاء تقييم كافة العناصر . اذا لم تكن متأكدا بشأن عنصر ما فالرجاء اعطاء افضل تقييم ممكن

	غير واثق تماما			واثق نوعا ما			واثق تماما		
1. المحافظة على الاعتماد على النفس	1	2	3	4	5	6	7	8	9
	غير واثق تماما			واثق نوعا ما			واثق تماما		
2. المحافظه على الموقف الايجابي	1	2	3	4	5	6	7	8	9
	غير واثق تماما			واثق نوعا ما			واثق تماما		
3. المحافظة على روح الدعابة	1	2	3	4	5	6	7	8	9
	غير واثق تماما			واثق نوعا ما			واثق تماما		
4. التعبيرعن المشاعرالخاصة بالمرض	1	2	3	4	5	6	7	8	9
	غير واثق تماما			واثق نوعا ما			واثق تماما		
5. التحدث عن ما يدوربفكري أحيانا	1	2	3	4	5	6	7	8	9
	غير واثق تماما			واثق نوعا ما			واثق تماما		
6. المحافظةعلى الانشطة (العمل,المنزل, الهوايات,الأمور الاجتماعية)	1	2	3	4	5	6	7	8	9
	غير واثق تماما			واثق نوعا ما			واثق تماما		
7. محاولة الهدوء اثناء العلاج وعدم السماح للافكار المخيفة ان تقلقني	1	2	3	4	5	6	7	8	9
	غير واثق تماما			واثق نوعا ما			واثق تماما		
8. المشاركة بفاعلية في قرارات العلاج	1	2	3	4	5	6	7	8	9
	غير واثق تماما			واثق نوعا ما			واثق تماما		
9. طرح اسئلة على الطبيب	1	2	3	4	5	6	7	8	9
	غير واثق تماما			واثق نوعا ما			واثق تماما		
10. السعي لطلب المسانده من العائله, الاصدقاء, المجموعات, والمنظمات	1	2	3	4	5	6	7	8	9
	غير واثق تماما			واثق نوعا ما			واثق تماما		
11. مشاركة مخاوفي وما يقلقني مع الاخرين	1	2	3	4	5	6	7	8	9
	غير واثق تماما			واثق نوعا ما			واثق تماما		
12. التحكم في القيء والغثيان سواء كنت اعاني من هذه المشاكل من قبل ام لا	1	2	3	4	5	6	7	8	9
	غير واثق تماما			واثق نوعا ما			واثق تماما		
13. مواجهة الصعوبات البدنية	1	2	3	4	5	6	7	8	9
	غير واثق تماما			واثق نوعا ما			واثق تماما		
14. محاولة الهدوء اثناء الانتظار لمدة ساعة واحدة على الاقل في موعد العلاج الخاص بي	1	2	3	4	5	6	7	8	9
